# Long-term outcomes of aortic valve insufficiency in patients with Takayasu arteritis

**DOI:** 10.3389/fimmu.2025.1667603

**Published:** 2025-09-22

**Authors:** Feyyaz Hazar Yağmur, Muhammet Emin Kutu, Fatih Tastekin, Aysegul Avcu, Senar Şan, Tuğba Ocak, Fatih Yıldırım, Hasan Kocaayan, Safiye Bakkal, Sema Işık, Burcu Ceren Uludoğan, Semiha Köroğlu, Gokce Kenar, Yavuz Pehlivan, Ayse Cefle, Ayten Yazıcı, Şule Yaşar Bilge, Servet Akar, Ahmet Omma, Fatoş Önen, Cemal Bes, Fatma Alibaz-Oner, Haner Direskeneli, Kenan Aksu, Gökhan Keser, Nilüfer Alpay Kanıtez

**Affiliations:** ^1^ Department of Internal Medicine, Koc University Faculty of Medicine, Istanbul, Türkiye; ^2^ Department of Internal Medicine, Division of Rheumatology, University of Health Sciences Doctor Sadi Konuk Training and Research Hospital, Istanbul, Türkiye; ^3^ Department of Internal Medicine, Division of Rheumatology, Ege University Faculty of Medicine, Izmir, Türkiye; ^4^ Department of Internal Medicine, Division of Rheumatology, Marmara University Faculty of Medicine, Istanbul, Türkiye; ^5^ Department of Internal Medicine, Division of Rheumatology, Kocaeli University Faculty of Medicine, Kocaeli, Türkiye; ^6^ Department of Internal Medicine, Division of Rheumatology, Uludag University Faculty of Medicine, Bursa, Türkiye; ^7^ Department of Internal Medicine, Division of Rheumatology, Basaksehir Cam and Sakura City Hospital, Istanbul, Türkiye; ^8^ Department of Internal Medicine, Division of Rheumatology, Izmir Katip Celebi University Faculty of Medicine, Izmir, Türkiye; ^9^ Department of Internal Medicine, Division of Rheumatology, Dokuz Eylul University Faculty of Medicine, Izmir, Türkiye; ^10^ Department of Internal Medicine, Division of Rheumatology, University of Health Sciences Ankara City Hospital, Ankara, Türkiye; ^11^ Department of Internal Medicine, Division of Rheumatology, Eskisehir Osmangazi University Faculty of Medicine, Eskisehir, Türkiye; ^12^ Department of Internal Medicine, Division of Rheumatology, Koc University Faculty of Medicine, Istanbul, Türkiye

**Keywords:** Takayasu Arteritis, aortic valve insufficiency, valvular involvement, echocardiography, prognosis, risk factors

## Abstract

**Objective:**

Takayasu arteritis (TAK) is a rare vasculitis predominantly affecting the aorta and its branches. Aortic valve (AV) insufficiency is a clinically significant but underexplored manifestation of TAK. This study aimed to evaluate the prevalence, associated risk factors, and prognostic implications of AV insufficiency in a large, multicenter cohort of TAK patients.

**Methods:**

We retrospectively analyzed data from 252 patients diagnosed with TAK across 11 rheumatology centers in Türkiye. Echocardiographic findings, clinical characteristics, treatment data, and long-term outcomes were collected. Severity and progression of AV insufficiency were assessed using standardized echocardiographic criteria.

**Results:**

AV insufficiency was present in 33% of patients, with moderate to severe forms observed in 12% and with 7% requiring valve replacement. Ascending aorta and renal artery involvement, hypertension, and heart failure were more frequent in patients with AV insufficiency. Over a median follow-up of 97 months, AV insufficiency progressed in 17% of baseline affected patients. The absence of subclavian artery involvement and vascular damage index (VDI) were found to be independent risk factors for AV insufficiency progression. Despite the structural progression, AV insufficiency did not significantly impact mortality, relapse rates, vascular damage index, or treatment patterns.

**Conclusion:**

AV insufficiency is the most clinically relevant valvular pathology in TAK, and its baseline presence did not adversely influence TAK disease outcomes. Progression was observed in a subset of patients, and this was associated with higher VDI scores, particularly among those without subclavian artery involvement. Timely diagnosis and multidisciplinary management may mitigate its impact on long-term outcomes.

## Introduction

Takayasu arteritis (TAK) is a rare, chronic, granulomatous vasculitis that primarily affects the aorta and its major branches and most commonly seen in young women and can lead to progressive arterial stenosis, occlusion, and aneurysm formation (1). The disease is often characterized by systemic inflammation, resulting in symptoms such as fatigue, fever, weight loss, and vascular complications, including diminished or absent pulses, hypertension, and ischemic manifestations and significant portion of patients with TAK experience cardiac valvular involvement, with the aortic valve being the most commonly affected (2). This involvement frequently results in valvular insufficiency, leading to progressive deterioration in valve function and negatively impacting disease outcomes such as heart failure (2). The involvement of the ascending aorta has been identified as a key risk factor for aortic valve (AV) insufficiency (3). Mitral valve (MV) insufficiency is not rare in TAK. However, given the relatively high prevalence of mild MV regurgitation in the general population and the possibility of MV abnormalities occurring secondary to aortic valve pathology, its clinical significance may be considered negligible (4). Although AV insufficiency in TAK is a clinically important manifestation, comprehensive data regarding its associated risk factors, the progression of AV insufficiency, and the long-term prognosis of affected patients remain limited. This study aims to investigate the frequency, risk factors, and prognosis of AV insufficiency in a large cohort of patients with TAK.

## Materials and methods

Subjects who were diagnosed with TAK according to the 1990 American College of Rheumatology criteria and had echocardiography performed at baseline, from eleven rheumatology centers in Türkiye were included in this retrospective longitudinal study. Patients with acute rheumatic fever and congenital cardiac valvular disease were excluded. Clinical, laboratory, echocardiography, computed tomography angiography and magnetic resonance angiography data were obtained from outpatient polyclinic files from January 2005 to May 2025. AV, MV, tricuspid valve (TV) and pulmonary valve (PV) abnormalities, including thickening, calcification, stenosis, and insufficiency of the valves, were defined. Mild, moderate, and severe valvular pathology were classified according to the 2017 AHA/ACC Focused Update of the 2014 AHA/ACC Guidelines for the Management of Patients with Valvular Heart Disease (5). Angiographic classification of involved arteries was based on Numano’s criteria (6). Vasculitis damage index (VDI), a validated damage score consisting of 63 parameters to assess various organ systems has been calculated throughout the patient visits (7). Total follow-up durations, number of relapses as well as VDI scores were collected from the last visit data. If there was deterioration in the degree of AV insufficiency or a new AV insufficiency detected on the last echocardiography, and/or AV intervention was performed, these conditions were defined as worsening in AV insufficiency.

Statistical analyses were performed using SPSS 21.0 (SPSS Statistics for Windows, Chicago: SPSS Inc.). Kolmogorov-Smirnov test was used to determine whether a continuous variable follows a normal distribution. Descriptive statistics are reported as the mean and standard deviation for variables with a normal distribution, while non-normally distributed variables are summarized using the median along with the interquartile ranges. Clinical findings were compared according to the presence of AV insufficiency. Differences between continuous variables and categorical data were tested using the student’s t-test or Mann-Whitney test and chi-square test. Those factors associated with AV insufficiency progression on univariate analyses at significance level p<0.2 were tested with multivariate analysis using logistic regression. All analyses used a 5% two-sided significance level and results were expressed as odds ratio (OR) and 95% confidence interval (CI).

The study was approved by the Ethics Committee of the University of Health Sciences Bakirkoy Dr. Sadi Konuk Training and Research Hospital on August 23, 2023, with decision number 2023-16-17.

## Results

A total of 252 patients with TAK were analyzed in our study. Median age of patients at diagnosis was 33.7 (20.9-46.5) years and 215 (85.3%) patients were females. MV insufficiency (42%) was the most commonly seen valvular abnormality and 16 (15%) of them had moderate to severe insufficiency (**Table 1**). Among the 83 patients (33%) diagnosed with AV insufficiency, 36% exhibited moderate or severe insufficiency. Among patients with moderate to severe valvular insufficiency, AV insufficiency (12%) was the most common, followed by MV insufficiency (7%). AV insufficiency was identified in 79 (75%) out of 106 patients diagnosed with MV insufficiency. Conversely, 15.8% of the patients without AV insufficiency exhibited only MV insufficiency. All patients with moderate or severe TV insufficiency had additional either AV and/or MV insufficiency.

**Table 1:** Valve involvement patterns on baseline echocardiograms.

Characteristics	Aortic valve	Mitral valve	Tricuspid valve	Pulmonary valve
Overall insufficiency	83 (33%)	106 (42%)	100 (40%)	9 (4%)
Moderate insufficiency	22 (9%)	12 (5%)	3 (1%)	0
Severe insufficiency	8 (3%)	4 (2%)	3 (1%)	0
Thickening	4 (2%)	7 (3%)	1 (0.5%)	1 (0.5%)
Stenosis	2 (1%)	2 (1%)	0	0
Calcification	10 (4%)	0	0	1 (0.5%)

Valve thickening was observed in a considerably smaller proportion of patients, occurring in 3% and 2% of cases with MV and AV involvement, respectively. Stenosis of either valve was identified in only 1% of the patients (**Table 1**).

The clinical and laboratory characteristics of patients with and without AV insufficiency are presented in **Table 2**. Ascending aorta (45%) and renal artery (37%) involvement were significantly more common among patients with AV insufficiency (p=0.049 and p=0.037, respectively). Additionally, the prevalence of hypertension (40%) and congestive heart failure (12%) was significantly higher in the group with AV insufficiency (p=0.020 and p=0.018, respectively). No statistically significant difference was observed between the two groups in terms of Numano radiological classification, sex, age, smoking and systemic findings.

**Table 2:** Baseline clinical features according to the presence of AV insufficiency.

Characteristics	AVI+ n:83	AVI – n:169	P values
Female n, (%)	72 (87%)	143 (85%)	1.000
Age at diagnosis (mean ± SD)	35.2 ± 12.8	33.0 ± 12.7	0.196
Delayed diagnosis (years, mean ± SD)	3.3 ± 5.3	2.5 ± 4.1	0.244
Smoker n, (%)	17 (20%)	31 (18%)	0.605
Vascular involvement n, (%)			
Ascending aorta	37 (45%)	52 (31%)	**0.049**
Descending aorta	31 (37%)	44 (26%)	0.106
Abdominal aorta	28 (34%)	49 (29%)	0.561
Carotid artery	54 (65%)	102 (60%)	0.674
Subclavian artery	66 (80%)	119 (70%)	0.212
Renal artery	31 (37%)	40 (24%)	**0.037**
Numano type n, (%)			
Type 1	19 (23%)	53 (31%)	
Type 2	18 (22%)	37 (22%)	
Type 3	3 (4%)	2 (1%)	
Type 4	1 (1%)	10 (6%)	
Type 5	41 (49%)	64 (38%)	0.131
Clinical findings n, (%)			
Systemic findings	30 (36%)	61 (36%)	0.775
Claudication	52 (63%)	95 (56%)	0.163
Peripheral murmur	17 (20%)	5 (3%)	0.859
Pulselessness	21 (25%)	51 (30%)	0.283
Hypertension	33 (40%)	47 (28%)	**0.020**
Coronary artery disease	6 (7%)	11 (7%)	0.325
Congestive heart failure	10 (12%)	6 (4%)	**0.018**
Cerebrovascular accident	6 (7%)	10 (6%)	0.174
Overlap autoimmune disease	6 (7%)	8 (5%)	0.349
CRP mg/l, (mean ± SD)	47.0 ± 47.94	40.2 ± 63.0	0.472
ESR mm/h, (mean ± SD)	49.7 ± 47.9	51.3 ± 33.2	0.734
Hyperlipidemia n, (%)	5 (6%)	24 (14%)	0.814

CRP, C reactive protein; ESR, Erythrocyte sedimentation rate; AVI, Aortic valve insufficiency.

Bold values are statistically significant.

The median follow-up duration was comparable between patients with and without AV insufficiency, calculated as 97.4±67.7 months and 98.8±64.8 months, respectively (p=0.873; **Table 3**). There were no statistically significant differences between the two groups in terms of relapse frequencies, vascular damage index scores, treatment regimens, or mortality rates (**Table 3**). A total of 21 patients were found to have progression of AV insufficiency during the follow-up period, including seven patients with newly developed AV insufficiency. Therefore, progression developed in 17% of our patients with baseline AV insufficiency, while regression of insufficiency was determined in 10% of those patients.

**Table 3:** Cumulative clinical findings and treatments according to the presence of baseline AV insufficiency.

Characteristics	AVI+ n:83	AVI – n:169	P values
Follow-up time (mean ± SD)	97.4 ± 67.7	98.8 ± 64.8	0.873
Relapse counts, median (IQR)	1 (0–2)	1 (0–2)	0.793
AVI progression	14 (17%)	7 (4%)	**0.001**
Cumulative cardiovascular event, n (%)			
Coronary artery disease	2 (3%)	5 (3%)	1.000
Congestive heart failure	4 (19%)	2 (8%)	0.765
Cerebrovascular accident	6 (9%)	10 (6%)	0.384
Cumulative prednisolone, g (mean ± SD)	8.9 ± 7.1	7.5 ± 7.4	0.309
Other treatments, n (%)			
Methotrexate	64 (77%)	107 (63%)	0.714
Azathioprine	35 (42%)	59 (35%)	1.000
Leflunomide	15 (18%)	29 (17%)	0.860
TNF inhibitors	31 (37%)	46 (27%)	0.288
Tocilizumab	15 (18%)	32 (19%)	0.726
Vascular damage index, median (IQR)	2 (1–4)	2 (0–3)	0.079
Death	8 (10%)	6 (4%)	0.080

AVI, Aortic valve insufficiency; IQR, interquartile range.Bold values are statistically significant.

Clinical features according to the progression of AV insufficiency are shown in **Table 4**. Six patients (%29) in the progression group subsequently underwent percutaneous or surgical aortic valve replacement (p=0.02). Abdominal aorta (52%) involvement was more frequent in the progression group, whereas subclavian artery (11%) involvement was less common, and VDI scores were higher among patients with progression (p values: 0.047, 0.016 and 0.001, respectively). Furthermore, absence of subclavian artery involvement and VDI emerged as independent risk factors for AV insufficiency progression in multivariate regression analysis (**Table 5**).

**Table 4:** Clinical features according to the presence of AV insufficiency progression.

Characteristics	AVI progression + n:21	AVI progression- n:231	P values
Female, n (%)	20 (95%)	194 (84%)	0.330
Follow-up time (mean ± SD)	94.1 ± 80.9	99 ± 64.2	0.790
Vascular involvement, n (%)			
Ascending aorta	7 (3%)	81 (35%)	1.000
Descending aorta	5 (2%)	70 (30%)	0.623
Abdominal aorta	11 (52%)	66 (29%)	**0.047**
Carotid artery	15 (71%)	140 (61%)	0.486
Subclavian artery	11 (52%)	173 (75%)	**0.016**
Renal artery	7 (3%)	64 (28%)	0.624
Numano type, n (%)			
Type 1	5 (2%)	67 (29%)	
Type 2	5 (2%)	50 (22%)	
Type 3	0 (0%)	5 (2%)	
Type 4	1 (5%)	10 (4%)	
Type 5	10 (48%)	94 (41%)	0.927
Hypertension, n (%)	11 (55%)	97 (55%)	1.000
Hyperlipidemia, n (%)	5 (25%)	32 (18%)	0.544
Cumulative cardiovascular event, n (%)			
Coronary artery disease	2 (12%)	12 (9%)	0.672
Congestive heart failure	1 (7%)	11 (10%)	1.000
Cerebrovascular accident	2 (10%)	14 (6%)	0.716
Cumulative prednisolone, g (mean ± SD)	11.3 ± 8.1	7.6 ± 7.2	0.151
Relapse counts, median (IQR)	1 (0–2)	1 (0–2)	0.889
Vascular damage index, median (IQR)	4 (3–4)	2 (0–3)	**0.001**
Aortic valve replacement	6 (29%)	0 (0%)	**0.020**
Death	0 (0%)	14 (6%)	0.615

AVI, Aortic valve insufficiency; IQR, interquartile range.Bold values are statistically significant.

**Table 5:** Multivariable analysis for AV insufficiency progression in TAK patients.

Characteristics	P value	OR	CI (95%)
Hyperlipidemia	0.380	0.533	0.131, 2.174
Congestive heart failure	0.266	1.778	0.646, 4.899
Carotid artery involvement	0.395	0.535	0.127, 2.258
Subclavian artery involvement	**0.009**	6.707	1.623, 27.727
Vascular damage index	**0.002**	1.557	1.174, 2.066

Bold values are statistically significant.

## Discussion

In our study, AV insufficiency stands out among other valvular pathologies in patients with TAK. Although MV and TV insufficiencies are more frequent in terms of prevalence, the proportion of moderate to severe involvement is particularly notable in AV insufficiency. TV insufficiency and MV insufficiency are rarely an isolated issue and are usually expected to develop secondary to AV insufficiency and the prevalence of mild MV insufficiency in the general population is reported to be around 10%, which is slightly higher than the prevalence observed among our patients without AV insufficiency (8). Therefore, as in previous studies, focusing on AV insufficiency in the clinical interpretation and management of cardiac valvular involvement in TAK patients appears to be appropriate (9, 10).

In three studies conducted in China, AV insufficiency was identified as the most prevalent valvular pathology among patients with TAK. Two of these studies, which involved a relatively small patient cohort, reported an AV insufficiency prevalence of nearly 50% (9, 11). The other, encompassing over 1,000 TAK patients, reported a frequency of 24%, with half of the cases classified as moderate or severe (12). Compared to that study, our study demonstrated a slightly higher overall prevalence of AV insufficiency; however, the proportion of moderate and severe cases was comparatively lower. All these rates are significantly higher than the prevalence of AV insufficiency observed in the general population. In the normal population, AV insufficiency ranks as the third most common valvular pathology and its prevalence increases with advancing age (13, 14). In the United States, the prevalence of moderate to severe AV insufficiency among individuals over the age of 75 is reported to be only 2% and the most well-established risk factors include AV stenosis and the presence of a bicuspid aortic valve (15).

According to the studies conducted by Zhang Y and Li J et al., the prevalence of MV insufficiency in patients with TAK was reported as 13%, which is lower than the rate observed in our patient cohort (3, 12). This difference may be due to the higher prevalence of other causes of MV insufficiency, such as acute rheumatic fever in our population (16). In addition, mitral valve prolapse which can progress to MV insufficiency, is common in the general population (17). Interestingly, they also reported TV insufficiency at a rate that was even lower than is typically observed in the general population (14). In our patient cohort, TV insufficiency was the second most common valvular pathology following MV insufficiency. However, clinically significant moderate to severe TV insufficiency was not observed in patients without concomitant AV or MV insufficiency, suggesting that TV involvement is predominantly secondary in nature.

The available literature on risk factors specifically associated with AV insufficiency in TAK remains quite limited. In most of the studies cited, risk assessments were conducted for overall valvular involvement rather than focusing on AV insufficiency alone. However, as discussed earlier, this approach is methodologically flawed. In the study by Shi et al., which specifically examined AV insufficiency, Numano type 2b angiographic classification, elevated C-reactive protein (CRP) levels, and ascending aorta involvement were identified as significant risk factors (9). Consistent with these findings, our study also suggests that ascending aorta involvement may be a contributing risk factor. A higher prevalence of heart failure findings in patients with AV insufficiency is expected (18). Furthermore, the frequent presence of renal artery involvement and hypertension in these patients warrants further investigation through comprehensive studies to clarify the causal relationship. It is plausible that hypertension contributes to AV insufficiency by increasing afterload (19).

There is a lack of significant data in the literature concerning the long-term follow-up and outcomes of TAK patients with AV insufficiency. In our study, although echocardiographic progression of AV insufficiency was observed in 17% of patients with a baseline diagnosis along with seven additional new diagnosis, this progression did not appear to significantly impact cardiovascular outcomes, number of relapses, cumulative prednisolone dose, or other treatment parameters. This stability may be attributed to effective medical management and timely interventional valve replacement. The increase in VDI appears to be closely related to AV insufficiency progression. Glucocorticoid-related damage may represent a determining factor at this stage. Nevertheless, it should be acknowledged that the VDI is not an ideal instrument for assessing damage in TAK. For instance, mechanical cardiovascular complications related to valvular pathology are also captured within the VDI. What adds to the complexity of interpretation is that cardiac valve involvement is directly incorporated into the VDI score. While ascending aortic involvement appears to act as a risk factor in the presence of baseline AV insufficiency, it does not seem to contribute to disease progression. By contrast, the protective effect of subclavian artery involvement demonstrated in our study warrants further investigation.

Six out of 30 patients with moderate to severe AV insufficiency underwent AV replacement during the follow-up period. Multidisciplinary monitoring of these patients in collaboration with cardiology and cardiovascular surgery departments is essential. In recent years, percutaneous AV replacement has become more common as an alternative to surgical intervention (20). However, there is currently no specific guideline addressing follow-up and replacement strategies for AV insufficiency in TAK patients. Notably, echocardiographic improvement was observed in 10% of patients, an outcome that differs from expectations in the general population and suggests that responsiveness to immunosuppressive therapy may influence decisions regarding valve replacement.

The most significant limitation of our study is the use of echocardiography, a subjective imaging modality, which was performed by different cardiologists at various centers, potentially introducing inter-observer variability. Furthermore, the retrospective design of the study restricted our capacity to obtain more comprehensive data on cardiovascular and echocardiographic outcomes. Additional limitations include the absence of a standardized follow-up protocol among participating centers and the lack of baseline echocardiographic assessments in all TAK patients, regardless of the presence or absence of cardiovascular symptoms.

In conclusion, our study is the first in the literature to provide comprehensive data on valvular involvement and disease prognosis in a substantial TAK patient cohort, and it identifies AV insufficiency as the predominant valvular pathology in patients with TAK. These patients exhibit high rates of ascending aortic involvement, hypertension, heart failure, and renal artery involvement. Furthermore, subclavian artery involvement is associated with a negative impact on the progression of AV insufficiency. Importantly, the presence of AV insufficiency does not adversely influence overall TAK disease outcomes. However, progression of AV insufficiency appears to be closely linked with VDI.

## Data Availability

The raw data supporting the conclusions of this article will be made available by the authors, without undue reservation.

## References

[1] Tombetti E Mason JC. Takayasu arteritis: advanced understanding is leading to new horizons. Rheumatol (Oxford). (2019) 58:206–19. doi: 10.1093/rheumatology/key040, PMID: 29635396

[2] Ren Y Du J Guo X Liu O Liu W Qi G. Cardiac valvular involvement of takayasu arteritis. Clin Rheumatol. (2021) 40:653–60. doi: 10.1007/s10067-020-05290-2, PMID: 32666179

[3] Li J Li H Sun F Chen Z Yang Y Zhao J. Clinical characteristics of heart involvement in Chinese patients with takayasu arteritis. J Rheumatol. (2017) 44:1867–74. doi: 10.3899/jrheum.161514, PMID: 28811356

[4] Santoro C Galderisi M Esposito R Buonauro A Monteagudo JM Sorrentino R. Global longitudinal strain is a hallmark of cardiac damage in mitral regurgitation: the Italian arm of the European Registry of mitral regurgitation (EuMiClip). Cardiovasc Ultrasound. (2019) 17:28. doi: 10.1186/s12947-019-0178-7, PMID: 31752893 PMC6873488

[5] Nishimura RA Otto CM Bonow RO Carabello BA Erwin JP Fleisher LA. 2017 AHA/ACC focused update of the 2014 AHA/ACC guideline for the management of patients with valvular heart disease: a report of the American College of Cardiology/American Heart Association Task Force on Clinical Practice Guidelines. Circulation. (2017) 135:e1159–95. doi: 10.1161/CIR.0000000000000503, PMID: 28298458

[6] Hata A Noda M Moriwaki R Numano F. Angiographic findings of Takayasu arteritis: new classification. Int J Cardiol. (1996) 54 Suppl:S155–63. doi: 10.1016/S0167-5273(96)02813-6, PMID: 9119518

[7] Exley AR Bacon PA Luqmani A Kitas GD Gordon C Savage S. Development and initial validation of the Vasculitis Damage Index for the standardized clinical assessment of damage in the systemic vasculitides. Arthritis Rheum. (1997) 40:371–80. doi: 10.1002/art.1780400222, PMID: 9041949

[8] Douedi S Douedi H. Mitral regurgitation. Treasure Island (FL: StatPearls Publishing (2025). Available online at: https://www.ncbi.nlm.nih.gov/books/NBK553135/. 31985928

[9] Shi X Du J Li T Gao N Fang W Chen S. Risk factors and surgical prognosis in patients with aortic valve involvement caused by Takayasu arteritis. Arthritis Res Ther. (2022) 24:102. doi: 10.1186/s13075-022-02788-9, PMID: 35526024 PMC9077813

[10] Fath AR Mookadam F Aglan A Eldaly AS Jahanyar J Shamoun F. Surgical management of aortic regurgitation in Takayasu’s arteritis: a systematic review of techniques and outcomes. Perm J. (2022) 26:103–13. doi: 10.7812/TPP/21.017, PMID: 35939573 PMC9676693

[11] Gao N Ci WP Tian CY Du J Zhou ZJ Wang T. Clinical data analysis of valvular involvement in takayasu arteritis. Zhonghua Yi Xue Za Zhi. (2016) 96:2138–41., PMID: 27464536 10.3760/cma.j.issn.0376-2491.2016.27.005

[12] Zhang Y Yang K Meng X Tian T Fan P Zhang H. Cardiac valve involvement in Takayasu arteritis is common: a retrospective study of 1,069 patients over 25 years. Am J Med Sci. (2018) 356:357–64. doi: 10.1016/j.amjms.2018.06.021, PMID: 30360804

[13] Andell P Li X Martinsson A Andersson C Stagmo M Zöller B. Epidemiology of valvular heart disease in a Swedish nationwide hospital-based register study. Heart. (2017) 103:1696–703. doi: 10.1136/heartjnl-2016-310894, PMID: 28432156 PMC5749343

[14] Singh JP Evans JC Levy D Larson MG Freed LA Fuller DL. Prevalence and clinical determinants of mitral, tricuspid, and aortic regurgitation (the Framingham Heart Study). Am J Cardiol. (1999) 83:897–902. doi: 10.1016/S0002-9149(98)01064-9, PMID: 10190406

[15] Nkomo VT Gardin JM Skelton TN Gottdiener JS Scott CG Sarano ME. Burden of valvular heart diseases: a population-based study. Lancet. (2006) 368:1005. doi: 10.1016/S0140-6736(06)69208-8, PMID: 16980116

[16] Demirbağ R Sade LE Aydın M Bozkurt A Acartürk E. The Turkish registry of heart valve disease. Turk Kardiyol Dern Ars. (2013) 41:1–10. doi: 10.5543/tkda.2013.71430, PMID: 23518931

[17] Otani K Takeuchi M Kaku K Haruki N Yoshitani H Eto M. Evidence of a vicious cycle in mitral regurgitation with prolapse: secondary tethering attributed to primary prolapse demonstrated by three-dimensional echocardiography exacerbates regurgitation. Circulation. (2012) 126:S214–21. doi: 10.1161/CIRCULATIONAHA.111.084178, PMID: 22965986

[18] Shahim B Shahim A Adamo M Chioncel O Benson L Crespo-Leiro MG. Prevalence, characteristics and prognostic impact of aortic valve disease in patients with heart failure and reduced, mildly reduced, and preserved ejection fraction: An analysis of the ESC Heart Failure Long-Term Registry. Eur J Heart Fail. (2023) 25:1049–60. doi: 10.1002/ejhf.2908, PMID: 37210639

[19] Rahimi K Mohseni H Kiran A Tran J Nazarzadeh M Rahimian F. Elevated blood pressure and risk of aortic valve disease: a cohort analysis of 5.4 million UK adults. Eur Heart J. (2018) 39:3596–603. doi: 10.1093/eurheartj/ehy486, PMID: 30212891 PMC6186276

[20] Avvedimento M Tang GHL. Transcatheter aortic valve replacement (TAVR): recent updates. Prog Cardiovasc Dis. (2021) 69:73–83. doi: 10.1016/j.pcad.2021.11.003, PMID: 34800439

